# Cryoablation in persistent atrial fibrillation – a critical appraisal

**DOI:** 10.1007/s12471-016-0858-y

**Published:** 2016-06-13

**Authors:** S. Tzeis, S. Pastromas, A. Sikiotis, G. Andrikopoulos

**Affiliations:** Pacing and Electrophysiology Department, Henry Dunant Hospital Center, Athens, Greece

**Keywords:** Persistent atrial fibrillation, Cryoballoon, Ablation

## Abstract

Ablation of atrial fibrillation is an established treatment for the management of patients with paroxysmal and persistent atrial fibrillation. The complex pathophysiology of persistent atrial fibrillation has fuelled the concept of adjunctive substrate modification on top of pulmonary vein isolation. However, recent studies have failed to demonstrate additive benefit from complex ablation approaches, thus supporting that standalone pulmonary vein isolation may prove sufficient, at least as the initial ablation strategy in persistent atrial fibrillation. In this premise, the new-generation cryoballoon is an attractive option in this demanding subgroup of patients due to its reliable efficacy in achieving pulmonary vein isolation combined with collateral debulking of the neighbouring atrial myocardium. In this review, we present a critical appraisal of the role of cryoablation in patients with persistent atrial fibrillation, discussing related technical considerations and existing scientific evidence.

## Introduction

Atrial fibrillation (AF) is the most common sustained cardiac arrhythmia and a major public health issue due to its increasing prevalence and its independent detrimental impact on patients’ quality of life and prognosis [1]. Based on data from the Framingham study, individuals older than 40 years, of both genders, have a 1 in 4 lifetime risk of developing AF [1]. The global prevalence of AF presents a rising trend mainly due to population ageing [2]. The projected burden of AF is estimated to increase substantially to more than double in the European Union from 2010 to 2060, with a profound effect on public health and health economics [3]. Therefore, optimisation of patient management and implementation of measures that may delay or prevent the natural course of this arrhythmia is of pivotal importance.

Atrial fibrillation has a rather predicted natural course characterised by an episodic pattern of increasing frequency and duration with time. The latest ESC and AHA/ACC/HRS guidelines have proposed classification of AF into paroxysmal, persistent and long-standing persistent types (Tab. 1; [4, 5]). The major difference is that the AHA/ACC/HRS guidelines rely solely on episode duration for AF classification irrespective of whether the index episode is terminated spontaneously or by intervention. On the other hand, the ESC guidelines categorise every AF episode terminated by cardioversion as persistent even if its duration is limited.

**Tab. 1:** Definition of paroxysmal, persistent and long-standing persistent atrial fibrillation based on the latest European and US guidelines

	ESC guidelines	AHA/ACC/HRS guidelines
Paroxysmal	Self-terminating episode, usually within 48 h but may last up to 7 days	Episode that terminates *spontaneously or with intervention* within 7 days of onset
Persistent	Episode lasting longer than 7 days *or terminated by cardioversion*	Continuous episode that is sustained longer than 7 days
Long-standing persistent	Episode lasting longer than one year	Continuous episode lasting more than 12 months

## Paroxysmal vs. persistent AF: pathophysiology and treatment practice

Paroxysmal AF differs from persistent AF not only in the duration of the arrhythmic episodes but also in the underlying mechanisms as well as the type and success rate of implemented ablation strategies. Paroxysmal AF is mainly associated with focal triggers or local reentries within or at the ostia of the pulmonary veins (PVs) [6, 7]. Therefore, the PVs traditionally constitute the only target of catheter ablation in paroxysmal AF patients, historically by ablating the intra-PV origin of the focal trigger, followed by segmental and currently by circumferential ablation at the antral level with verification of electrical isolation of all PVs [8, 9]. PV isolation can be achieved by different energy sources, the most widely used being cryoenergy and radiofrequency energy [9–11]. The FIRE and ICE trial, the largest randomised trial to compare the efficacy and safety of cryoballoon ablation and point-by-point radiofrequency current ablation in patients with paroxysmal AF, has been published recently [12]. Based on the study findings, PV isolation by cryoballoon ablation is associated with similar efficacy and safety as compared with radiofrequency ablation in paroxysmal AF patients [12].

The pathophysiology of persistent AF is shifted progressively from the PVs (triggers) to the atrial myocardium (substrate) through a complex process involving diverse mechanisms such as electrical, structural remodelling and atrial fibrosis [13]. This mechanistic insight, in conjunction with the suboptimal efficacy of PV isolation (PVI) alone in persistent AF ablation, provided the concept of substrate modification as an additive ablation technique [9, 14]. Several adjunctive ablation targets have been proposed, including linear lesions [15], complex fractionated atrial electrograms (CFAEs) [16], stable rotors or focal sources [17], low-voltage areas in the left atrium [18], sites of high dominant frequency [19], and ganglionated plexi [20]. Interestingly, the HRS/EHRA/ECAS expert consensus statement on catheter ablation of AF recommends a more extensive ablation including linear lesions and complex fractionated electrograms when treating patients with long-standing persistent AF [9]. However, it should be emphasised that this consensus recommendation is not based on solid evidence, since the pertinent trials are limited mainly by a small sample size [15, 21, 22]. Interestingly, a recently published survey aiming to assess real-world, everyday practice among 30 European centres reported that 67 % of them use only standalone PVI as a first-ablation technique in persistent AF patients [23]. This finding shows that the electrophysiological community has not yet been persuaded that the implementation of adjunctive substrate modification on top of PVI during persistent AF ablation has a favourable impact on patient outcome, with an acceptable risk-to-benefit ratio.

The practice of standalone PVI for persistent AF ablation is expected to be further reinforced by the findings of the STAR-AF 2 trial, the largest ever multicentre study comparing ablation strategies in this type of AF patient population. STAR-AF 2 randomised 589 patients with persistent AF (three-quarters in constant AF for more than 6 months) in a 1:4:4 ratio to PVI, PVI plus CFAE ablation and PVI plus lines [24]. Apart from the sample size, additional methodological strengths of the study should be mentioned. Patient follow-up was long (18 months) with rigorous rhythm monitoring (systematic weekly transtelephonic monitoring and in case of symptoms), patients were unaware of the implemented ablation technique and a second procedure was allowed at 3–6 months but only by repeating the same ablation strategy in which the patient was initially randomised (avoiding crossover). Furthermore, acute procedural endpoints were achieved at high rates in all ablation groups (97 % successful PVI, complete conduction block across both lines in 74 % of patients and inability to eliminate all CFAEs in only 9 %), suggesting that almost maximal benefit was gained from the adopted ablation techniques. The percentage of patients free from arrhythmia recurrence was similar in the three groups (PVI: 59 %, PVI+CFAE: 49 % and PVI plus lines: 46 %, *p* = 0.15), without any difference after two ablation procedures. In addition, fluoroscopy exposure and procedure time were significantly shorter in the PVI group.

It should be noted that although ablation has earned its evidence-based credentials for symptomatic improvement of patients with AF, the results of ongoing trials (CABANA and EAST trials) are expected to shed light on the potential effect of this invasive treatment on hard endpoints such as stroke incidence and mortality [9].

## Technical features of cryoballoon

**Fig. 1:**
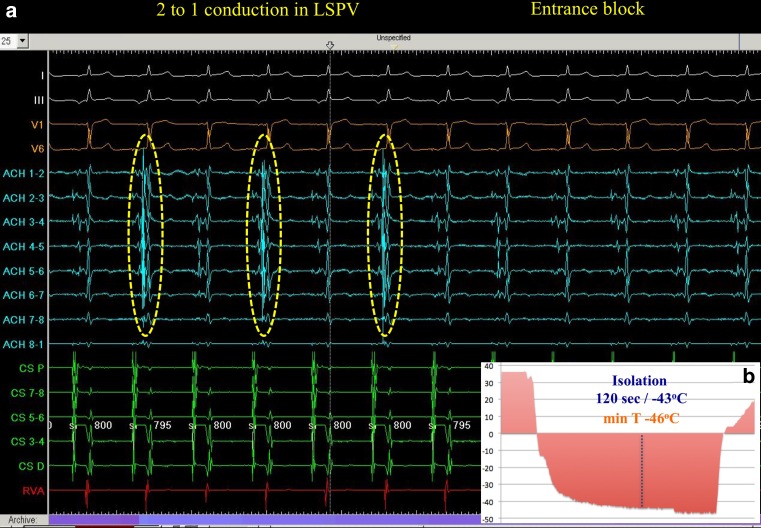
Isolation of the left superior pulmonary vein (LSPV) using the second-generation cryoballoon in a patient with persistent atrial fibrillation. **a** In the left half of the tracing, there is 2 to 1 entrance block in the LSPV (the dotted yellow ovals demarcate PV potentials). In the right half of the recording, the PV potentials disappear, documenting the achievement of entrance block. **b** Plot of freezing temperature over time (lesion duration 240 sec). Isolation was achieved in 120 sec at a temperature of −43 ^o^C, while the nadir temperature achieved was −46 ^o^C

**Fig. 2:**
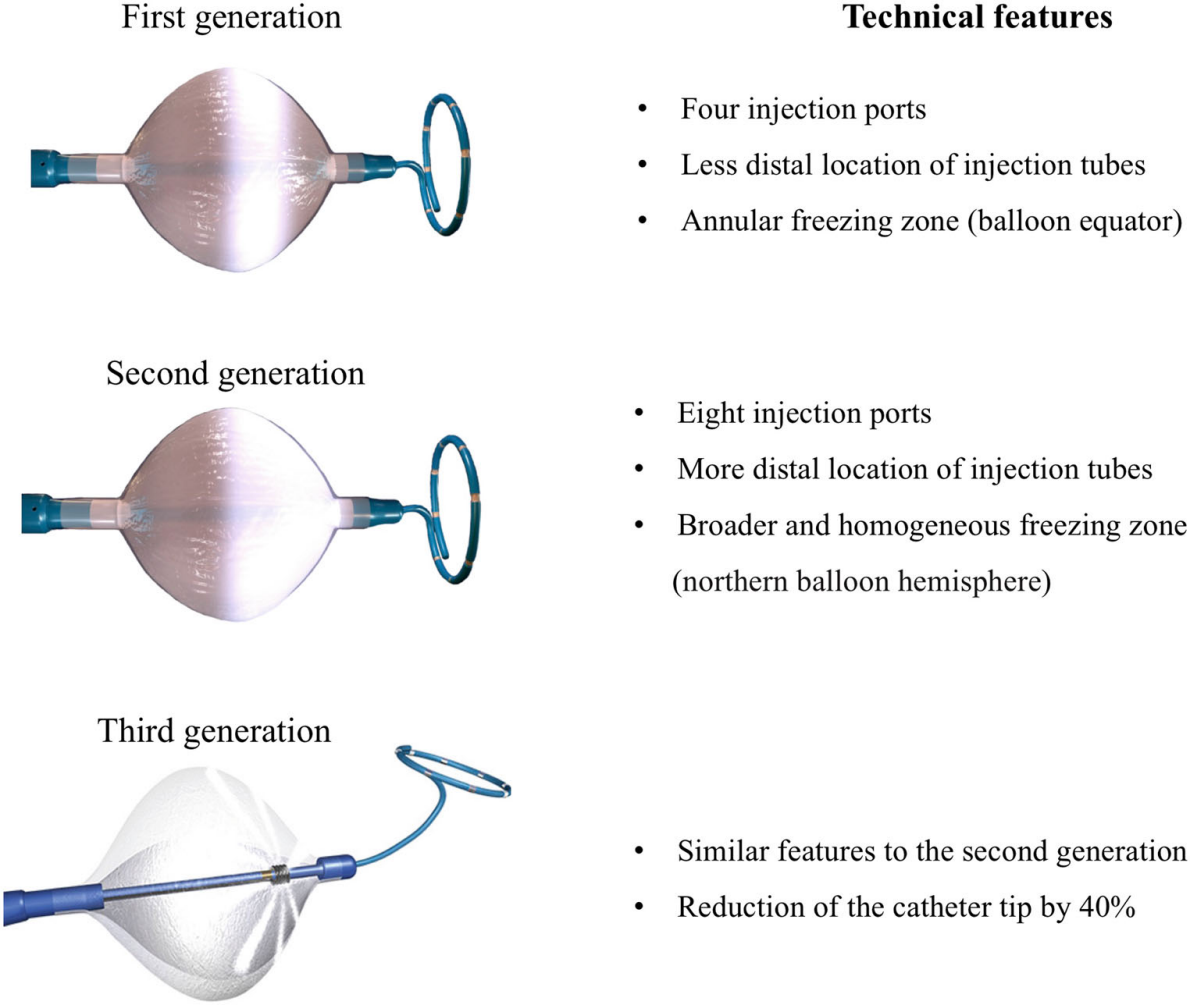
Different generations of cryoballoons with respective technical features

Cryoablation of AF incorporates an expandable balloon-based catheter system, which is placed at the entrance of each PV and enables the electrical disconnection between the PVs and left atrium (Fig. 1). However, the performance of the first-generation cryoballoon (Medtronic, Inc., Minneapolis, MN) was suboptimal since additional focal ablation was necessary in up to one-third of patients in order to achieve electrical PVI [25]. This caveat was at least partly attributed to the inhomogeneous and limited distribution of the freezing zone only around the equator of the balloon (Fig. 2; [26]). Therefore, in order to achieve contact of the freezing zone with the targeted myocardium, the first-generation cryoballoon had to be placed at the PV ostium with a strictly coaxial orientation, which proved demanding especially in peculiar PV anatomies and in the right inferior PV.

The second-generation cryoballoon, approved by the US Food and Drug Administration (FDA) in 2012, incorporated several technical improvements, including eight injection tubes, compared with four in the first-generation cryoballoon, and a more distal location of the injection ports on the catheter shaft. These modifications enable a larger and more uniform freezing zone covering the entire northern hemisphere of the balloon [26]. From a practical perspective, this cooling feature facilitates the procedure by enabling contact of the ice cap with the PV antrum, even if the catheter balloon shaft is not parallel to the targeted vein. In addition it enhances proper contact of the ice cap even if the PV antrum has an uncommon shape. These features have increased procedural efficacy and have resulted in significantly higher freedom from AF recurrence when using second- as compared with first-generation cryoablation [27–30]. However, several studies have reported an increased occurrence rate of phrenic nerve palsy in comparison with first-generation cryoballoon, which is possibly associated to the more extended freezing area of the second cryoballoon [27].

In May 2015, a third-generation cryoballoon (Arctic Front Advance® ST) received approval from the FDA for the treatment of patients with drug refractory, recurrent, symptomatic, paroxysmal AF. The difference in catheter design in comparison with the second-generation cryoballoon is the 40 % reduction in the length of the catheter tip. This feature provides improved manoeuvrability and allows a more proximal retraction of the circular catheter during ablation and thus a higher rate of real-time PV recordings [31]. Real-time monitoring of PV potentials is important to document the time of isolation (time to effect); this procedural parameter is a predictor of permanent PVI and should be taken into account to tailor the ablation strategy and the freeze duration. In a recently reported practice guide, a reduction of cryolesion duration to 150 seconds is recommended in case of early time to effect (≤30 sec) [32].

## Studies of cryoablation in persistent atrial fibrillation

**Fig. 3:**
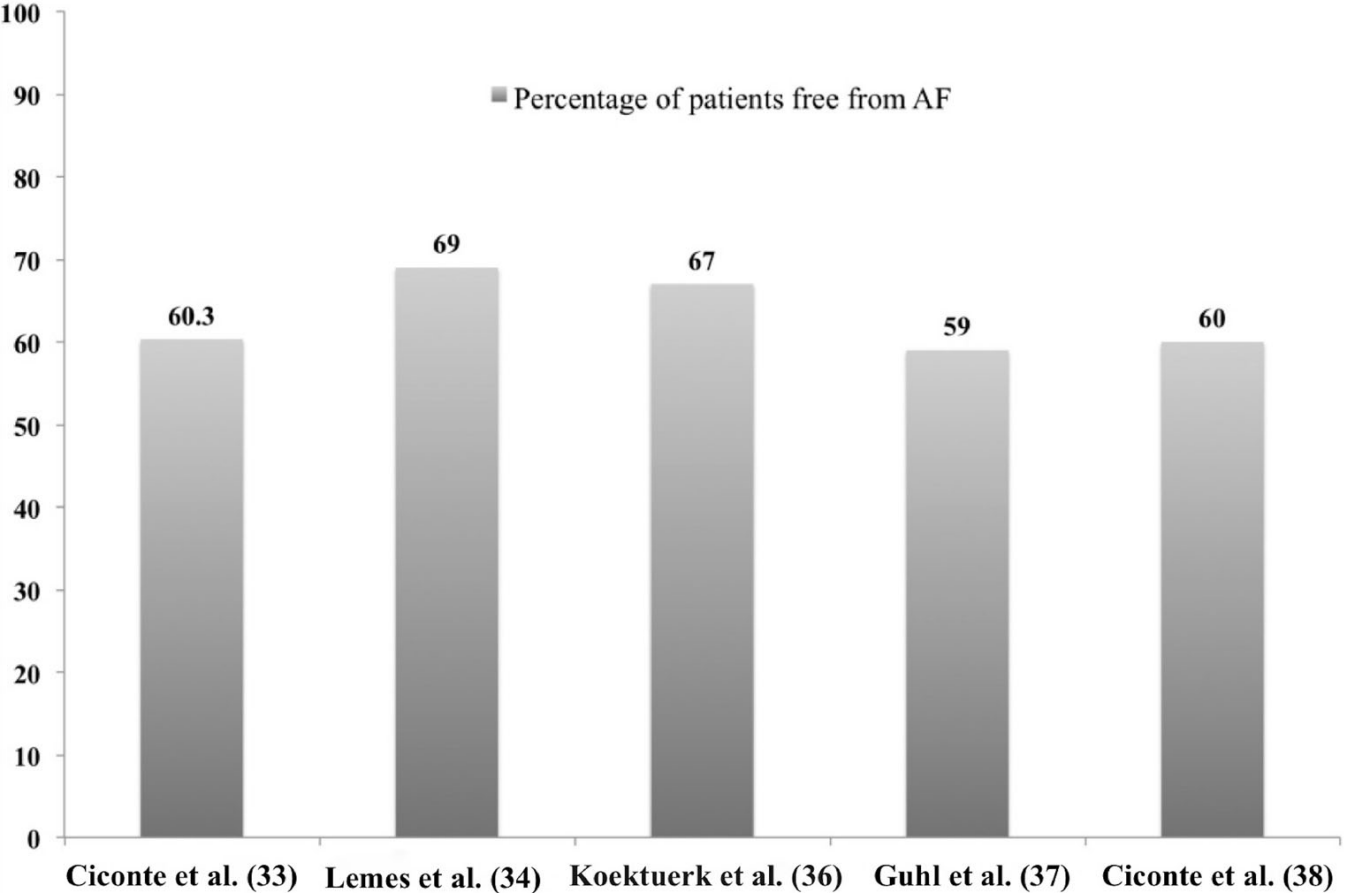
Reported freedom from atrial fibrillation during a follow-up period of 12 months following cryoablation of persistent atrial fibrillation (taking into account a 3-month blanking period). The reported percentage of the study by Koektuerk et al. pertains to a follow-up period of 10.6 ± 6.3 months

Based on the aforementioned ‘less is more’ approach in persistent AF ablation, PVI may prove to be a sufficient ablation strategy in persistent AF, since adjunctive substrate modification seems to provide no additional favourable impact. Therefore, cryoablation of the PVs may also suffice not only in paroxysmal but also in persistent AF. This hypothesis has been tested in several recent studies (Tab. 2, Fig. 3).

**Tab. 2:** Cryoablation studies (published as full papers) exclusively in persistent AF patients: methodological characteristics and acute procedural outcomes

Study	N	Balloon type	Procedure duration (min)	Fluoroscopy time (min)	Acute PVI/PNP rate in %	Comments
Ciconte et al. [33]	63	28 mm-CB2 (100 %)	87.1 ± 38.2	14.9 ± 6.1	100/6.3	First study on persistent AF No CMAP
Lemes et al. [34]	49	28 mm-CB2 (100 %)	113.6 ± 33.5	21.3 ± 6.7	100/0	Post-blanking continuation of AAD in 33 % of patients
Koektuerk et al. [36]	100	28 mm-CB2 (100 %)	96.2 ± 21.3	19.7 ± 6.7	100/3	Bonus 240-sec freeze post-PVI in all patients
Guhl et al. [37]	69	CB2 (88.4 %)	147 ± 45	45.0 ± 20.2	100/9	17 % of AF-free patients at one year were still on AADs CMAP in a subset of patients
Ciconte et al. [38]	100^a^	28 mm-CB2 (100 %)	90.5 ± 41.7	14.5 ± 6.6	100/4	First comparison of cryo vs. RF in persistent AF patients

*N* number of patients included, *PVI* pulmonary vein isolation, *PNP* phrenic nerve palsy, *CB2* second-generation cryoballoon, *CMAP* compound motor action potential, *AAD* antiarrhythmic drugs, *AF* atrial fibrillation, *RF* radiofrequency

^a^Total patient population – 50 patients in the cryoablation group

Ciconte et al. reported the first study of cryoablation in 63 consecutive, prospectively evaluated patients with persistent AF (mean duration of continuous AF persistence 7.2 months) [33]. Acute PVI was achieved in all PVs with a 28 mm second-generation cryoballoon, without the need for any additional focal lesions. During ablation, 27 % of patients with AF at presentation converted to sinus rhythm. Phrenic nerve palsy occurred in 6.3 % of patients and persisted post-discharge in only one patient. Freedom from any tachyarrhythmia after a single ablation, taking into account a 3-month blanking period, was 60.3 %. Furthermore, relapses during the blanking period and duration of persistent AF were independent predictors of arrhythmia recurrence during follow-up. This last finding argues in favour of ablation at earlier stages in the natural course of persistent AF.

In a retrospective study of 48 persistent AF patients, Lemes et al. reported a 100 % acute PVI rate and a 1-year clinical success rate of 69 % [34]. Bonus cryolesion after successful PVI was omitted systematically after the initial minority of patients, in an effort to minimise potential collateral damage of cumulative cryoenergy in the phrenic nerve and the oesophagus. Furthermore, the combination of intermittent fluoroscopy during freezing, tactile feedback of diaphragmatic contraction and monitoring of continuous motor action potential resulted in no phrenic nerve palsy [35]. Electrical reconnection of previously isolated PVs was documented in the majority of patients with atrial tachyarrhythmia recurrence.

Koektuerk et al. evaluated the performance of cryoablation in a cohort of 100 patients with persistent AF (mean duration 5.5 ± 3.7 months) [36]. In a mean follow-up period of 10.6 ± 6.3 months, 67 % of patients were free from atrial tachyarrhythmia recurrence when considering a 3-month blanking period. Phrenic nerve palsy occurred in three patients despite recording of diaphragmatic compound motor action potential during phrenic nerve pacing, but it resolved during the intervention in two of them. All patients experienced a significant improvement in their EHRA scores, irrespective of the ablation outcome, though the magnitude of symptomatic improvement was significantly increased among patients without atrial tachyarrhythmia recurrence. Atrial tachyarrhythmia recurrence during the blanking period was the only significant independent predictor of recurrence at the end of the follow-up period. In a single-centre, prospective registry of consecutive persistent AF patients, Guhl et al. reported a 1-year, single-procedure, atrial arrhythmia recurrence-free rate of 59 %, taking into account a 3-month blanking period. In this case series the second-generation cryoballoon was used in 88.4 % of patients and the reported rate of major complications was 2.8 % [37].

To our knowledge, only one study has compared radiofrequency versus cryoablation exclusively in persistent AF patients [38]. In this single-centre, non-prospective, non-randomised study, the single procedural outcome of radiofrequency ablation (using three-dimension mapping and contact-force ablation catheter) versus cryoablation (28 mm second-generation cryoballoon) was assessed in a cohort of 100 patients with symptomatic, drug-refractory, persistent atrial fibrillation (mean duration of longest continuous time spent in AF 7.2 and 7.6 months respectively). Procedural duration and fluoroscopy time were significantly shorter in the cryoablation group. The percentage of patients free from all documented atrial tachyarrhythmias lasting more than 30 sec, without antiarrhythmic drugs and following a single ablation procedure, was similar between the compared groups after 12 months of follow-up (60 % with cryoablation versus 56 % with radiofrequency ablation, *p* = 0.71, when a 3-month blanking period was taken into account). In multivariate analysis, duration of persistent AF and relapse during the blanking period were the only significant independent predictors of arrhythmia recurrences.

Several ongoing trials are expected to shed further light in the efficacy and safety of cryoablation in patients with persistent atrial fibrillation (Tab. 3).

**Tab. 3:** Ongoing trials aiming to evaluate the role of catheter ablation with cryoballoon in persistent atrial fibrillation. (ClinicalTrials.gov April 2016)

Name of the trial	AF type studied	Identifier	Status
Cryoballoon Ablation in Patients With Longstanding Persistent Atrial Fibrillation (CRYO-LPAF)	Longstanding persistent	NCT02294929	Recruiting
Persistent Atrial Fibrillation Cryoballoon Ablation (PAFCA)	Persistent	NCT02166723	Active, not recruiting
A Prospective Study of Medical Therapy Against Cryoballoon Ablation in Symptomatic Recent Onset Persistent AF (METACSA)	Early onset persistent	NCT02389218	Not yet recruiting
Cryoballoon vs. Irrigated Radiofrequency Catheter Ablation: Double Short vs. Standard Exposure Duration (CIRCA-DOSE)	Paroxysmal or early persistent	NCT01913522	Recruiting
Catheter Ablation Compared With Pharmacological Therapy for Atrial Fibrillation (CAPTAF)	Paroxysmal/persistent	NCT02294955	Active, not recruiting
Cryoballoon Ablation for Early Persistent Atrial Fibrillation (Cryo4 Persistent AF)	Early persistent	NCT02213731	Recruiting
FREEZE Cohort Study	Paroxysmal/persistent	NCT01360008	Active, not recruiting
Prospective, Randomized Comparison of Hybrid Ablation vs. Catheter Ablation (PRHACA)	Persistent	NCT02344394	Recruiting

## Strengths and limitations of the cryoballoon in persistent AF ablation

The anatomy of the pulmonary veins presents considerable variation in shape, size and branching pattern. The existing discrepancy in the reported PV diameters is attributed to differences in the imaging modalities used, in the studied patient populations but mainly to the lack of a practical consensus definition of the level of the antrum, which separates the PVs from the left atrium [39–42]. A consistent finding in all studies is the oval shape of the PVs, especially of left PVs, with the minimal cross-sectional diameter oriented in the anteroposterior direction [39, 40]. Furthermore, in a large series of 473 consecutive patients who underwent contrast-enhanced magnetic resonance imaging of the left atrium and PVs, the diameters of the PVs were larger in patients presenting with persistent as compared with paroxysmal AF [39]. Even when the highest values of the maximal PV diameters among persistent AF patients are taken into account (ranging from 20.1 to 22.9 mm), they were considerably less than the critical value of 28 mm, which is the diameter of the spherically-shaped cryoballoon used in everyday practice.

Due to this area mismatch between balloon and PVs, when the cryoballoon is positioned against the PV antrum, its cooling distal hemisphere comes in contact not only with the PV antra but also with adjacent atrial myocardial tissue. It is worth noting that since the anteroposterior diameter of the veins is typically smaller than their superior-inferior diameter, the collaterally ablated atrial tissue is located mostly at the posterior and anterior wall. Kenigsberg et al. elegantly calculated the area of the ablated cardiac tissue after cryoablation of the PVs by performing a post-cryoablation electroanatomical voltage map of the left atrium [43]. In total, only 27 % of the entire left atrial posterior wall surface area remained electrically intact and unablated following cryoablation with a 28-mm cryoballoon.

Therefore, it is of primary importance that although the cryoballoon conceptually only targets the PVs, it additionally performs considerable electrical debulking of the left atrium. This widely circumferential extension of the cooling area may provide collateral benefit by ablating local contributors in AF triggering and maintenance such as ganglionic plexi and rotors, which may have therapeutic implications among patients with persistent AF [17, 44, 45].

On the other hand, the inadvertent extension of the cooling area at the posterior wall raises concern regarding potential collateral damage of the neighbouring oesophagus. The incidence of induced oesophageal thermal lesions with the use of the second-generation 28-mm cryoballoon in paroxysmal and persistent AF ablation is 12 % and is significantly correlated with the lowest endoluminal oesophageal temperature during each freeze cycle [46]. The interruption of cryoenergy delivery when the lowest endoluminal oesophageal temperature reaches a certain cut-off limit has been proposed as a safety measure to reduce the occurrence of oesophageal lesions [47]. From a practical viewpoint, a cut-off oesophageal temperature limit of 15 ^o^C is associated with a very low (1.5 %) incidence of oesophageal injury without impairing the achievement of PVI [47].

A detailed description of the advantages and pitfalls of using cryoablation for persistent AF ablation is presented in Tab. 4.

**Tab. 4:** Pros and cons of cryoablation in persistent atrial fibrillation

*Advantages* Reliable acute pulmonary vein isolation (PVI) in practically all patients Efficacy beyond PVI due to extended cooling area in the left atrium with significant posterior wall electrical debulking Easy-to-use and less cumbersome compared with point-by-point circumferential ablation No need for three-dimension mapping system Reduced procedure and fluoroscopy time compared to classical ablation strategies
*Disadvantages* Inability to target ad-hoc extra-pulmonary vein triggers or factors contributing to AF maintenance (e. g. rotors, ganglionated plexi), as is the case especially with redo procedures Inability for additional substrate modification (if and when needed!) Difficulties in peculiar anatomies (common trunk, atypical branching patterns) Inability to ablate ad-hoc atrial macro-reentry tachycardias Additional catheter needed to perform pacing manoeuvers for ruling out far-field recording of left atrial appendage electrograms in the circular catheter when targeting the left veins Potential collateral oesophageal injury Incidence of phrenic nerve palsy, although reduced when safety precautions implemented

## Conclusion

Invasive treatment of persistent AF is a challenging goal due to its diverse pathophysiology. Until novel mechanistic insights enable a more tailored and individualised approach to persistent AF patients, standalone PVI may be sufficient, at least as the initial invasive ablation strategy. In this line, the new-generation cryoballoon seems to be an easy-to-use treatment option, which provides reliable PVI with adjunctive debulking of the neighbouring atrial myocardium. Accumulating experience and data from upcoming studies is expected to further elucidate the role of cryoablation in the management of patients with persistent atrial fibrillation.
